# Stopping of Mycophenolic Acid in Kidney Transplant Recipients for 2 Weeks Peri-Vaccination Does Not Increase Response to SARS-CoV-2 Vaccination—A Non-randomized, Controlled Pilot Study

**DOI:** 10.3389/fmed.2022.914424

**Published:** 2022-06-10

**Authors:** Florina Regele, Andreas Heinzel, Karin Hu, Lukas Raab, Farsad Eskandary, Ingrid Faé, Sieglinde Zelzer, Georg A. Böhmig, Gregor Bond, Gottfried Fischer, Rainer Oberbauer, Roman Reindl-Schwaighofer

**Affiliations:** ^1^Division of Nephrology and Dialysis, Department of Internal Medicine III, Medical University of Vienna, Vienna, Austria; ^2^Department of Blood Group Serology and Transfusion Medicine, Medical University of Vienna, Vienna, Austria; ^3^Clinical Institute of Medical and Chemical Laboratory Diagnostics, Medical University of Graz, Graz, Austria

**Keywords:** SARS-CoV-2, kidney transplantation, immunosuppressant, mycophenolate, azathioprine

## Abstract

**Introduction:**

Kidney transplant recipients (KTR) are at high risk of developing severe COVID-19. However, vaccine response in this population is severely impaired with humoral response rates of 36–54 and 55–69% after two or three doses of SARS-COV-2 vaccines, respectively. Triple immunosuppression and specifically the use of anti-proliferative agents such as mycophenolic acid (MPA) or azathioprine (AZA) have been identified as risk factors for vaccine hypo-responsiveness.

**Methods:**

We hypothesized that in vaccine non-responders to at least three previous vaccine doses, pausing of MPA or AZA for 1 week before and 1 week after an additional vaccination would improve humoral response rates. We conducted an open-label, non-randomized controlled pilot study including 40 KTR with no detectable humoral response after three or four previous vaccine doses. Primary endpoint was seroconversion following SARS-CoV-2 vaccination. MPA and AZA was paused in 18 patients 1 week before until 1 week after an additional vaccine dose while immunosuppression was continued in 22 patients.

**Results:**

There was no difference in the humoral response rate between the MPA/AZA pause group and the control group (29 vs. 32%, *p* > 0.99). Absolute antibody levels were also not statistically significantly different between the two groups (*p* = 0.716).Renal function in the MPA/AZA pause group remained stable and there was no detection of new onset donor-specific antibodies or an increase of donor-derived cell-free DNA serving as a marker of allograft damage throughout the study period.

**Conclusion:**

Pausing of MPA/AZA for 2 weeks peri-vaccination did not increase the rate of seroconversion in kidney transplant. However, one in three KTR without humoral immune response to at least three previous vaccinations developed antibodies after an additional vaccine dose supporting continued vaccination in non-responders.

## Introduction

Kidney transplant recipients (KTR) are at an increased risk of developing severe forms of COVID-19 due to therapeutic immunosuppression and comorbidities (1). Previous studies showed a severely impaired immune response to various vaccination protocols using both mRNA- and vector-based vaccines. Seroconversion rates following two vaccine doses ranged between 36 and 54% primarily depending on immunosuppressive medication. Subsequent third vaccination in KTR who remained negative after the first two vaccine doses results in a detectable humoral immune response in another 30–40%. Antibody-levels in responders are also significantly lower compared to the general population (2–6). Consequently, despite vaccination, KTR remain at high risk for severe COVID-19 with hospitalization and mortality rates of 40 and 22%, respectively (7, 8).

Risk factors for vaccine non-response are primarily related to the level of immunosuppression in KTR. Triple immunosuppressive therapy and in particular the use of belatacept or mycophenolic acid (MPA) have been identified as risk factor for vaccine non-response (9). MPA is a reversible inhibitor of the inosine monophosphate dehydrogenase (IMPDH), an enzyme essential to the *de novo* synthesis of guanosine-triphosphate (GTP) in lymphocytes (10). Azathioprine (AZA) is used as alternative to MPA in KTR has a similar anti-proliferative effect on lymphocytes (11).

Short term discontinuation of antiproliferative immunosuppression has been proposed as strategy to increase response to SARS-CoV-2 vaccines in immunosuppressed individuals (12). Several studies suggest that temporarily withholding MPA in KTR may be safe and does not increase acute rejection episodes (13). However, risk for long-term allo-sensitization and development of donor specific antibodies (DSA) has to be balanced against potential benefits of vaccine response.

We hypothesized that temporary suspension of MPA or AZA for 2 weeks peri-vaccination will increase seroconversion in KTR without a humoral immune response to three or more SARS-CoV-2 vaccine doses.

## Methods

### Study Design

This was a non-randomized, open label, controlled pilot study investigating the efficacy and safety of withholding MPA or AZA 1 week before until 1 week after a SARS-COV2-vaccination with BNT162b2 in KTR, who had no anti-SARS-CoV2 spike protein antibodies after at least three previous SARS-CoV2 vaccine doses (i.e., <0.8 BAU/mL). The trial was conducted from November 15th 2021 to January 15th 2022, at the outpatient clinic of the Department of Nephrology and Dialysis at the Medical University of Vienna (Austria). The trial was approved by the ethics committee of the Medical University of Vienna (1612/2021). All patients gave written informed consent before participating in the study.

### Study Cohort

The study cohort comprised adult kidney transplant recipients, who had no SARS-CoV2 spike protein antibodies at least 4 weeks after a 3rd or 4th SARS-CoV2 vaccine dose. There was no limitation with respect to the type of vaccines (mRNA or vector) previously administered. Only patients with a triple immunosuppressive therapy containing MPA or AZA were included. Patients with a prior documented SARS-CoV-2 infection were excluded.

### Procedures

After obtaining written informed consent, patients were evaluated for stopping maintenance therapy with MPA or AZA. Decision on MPA/AZA pause was based upon individual risk assessment by a transplant physician and patient preference: Specifically, MPA/AZA was not reduced in KTR with a history of biopsy-proven chronic rejection or known donor-specific antibodies (DSA) as well as patients who preferred additional vaccination without stopping of MPA/AZA treatment. Patients in the immunosuppression reduction arm stopped their maintenance therapy with MMA/AZA on day 1. On day 8, patients received a 4th/5th dose of the BNT162b2 SARS-CoV2 vaccine. Patients withholding MPA/AZA were followed-up on day 14 for a visit to assess safety parameters. MPA/AZA was restarted on day 15 at the previously prescribed dosage. Assessment of the primary endpoint (i.e., SARS-CoV2 spike protein antibodies) was scheduled at 4 weeks (28–35 days) after vaccination.

### Outcomes

The primary outcome was the rate of seroconversion, defined as the number of patients with SARS-CoV-2 spike protein antibody levels > 0.8 U/mL determined by Elecsys^®^ at 4 weeks after vaccination. Secondary outcomes were the level of SARS-CoV-2 spike antibodies and number of sero-converted patients exceeding 264 BAU/mL, a threshold associated with 80% vaccine efficacy against symptomatic COVID-19 infection caused by the SARS-CoV-2 alpha variant (14).

In order to evaluate the safety of pausing MPA/AZA we assessed serum creatinine, donor-specific antibodies (DSA) and donor-derived cell-free (dd-cfDNA) at beginning and end of the MPA pause, as well as 4 weeks after vaccination. We also measured the plasma load of torque-teno virus (TTV) as a surrogate for the overall immunosuppression, as well as plasma levels of mycophenolic acid to assess compliance.

### Laboratory Measurements

#### Antibody Response Against SARS-CoV-2

Antibody response was evaluated using the Elecsys^®^ Anti- SARS-CoV-2 enzyme immunoassay (RocheDiagnostics), which tests for the receptor-binding domain of the SARS-CoV-2 spike protein (cutoff, ≥0.8 U/mL; according to manufacturer's instructions). Samples with levels below the limit of detection were set to 0.2 U/mL. The measured U/mL are highly correlated with the World Health Organization's International Standard BAU/mL (*r* = 0.9996; U/mL = 0.972 × BAU/mL; per manufacturer's instructions).

#### Cell-Free Donor Derived DNA

Initially, donor and recipient were genetically discriminated with genomic markers (KMR Typing Kit, GenDX Utrecht). Cell free DNA was isolated from plasma and donor specific DNA content was monitored using qPCR for a housekeeping gene and an informative genomic marker. We have previously validated 1% as lower level of detection (15).

#### Donor-Specific Antibodies

For HLA antibody detection, single-antigen flow-bead assays (One Lambda, Canoga Park, CA) were used as previously described (16). An MFI threshold >1000 was considered as positive.

#### TTV

Torque teno virus (TTV) DNA was quantitated from 200 μL of plasma by real-time polymerase chain reaction, as previously described (17). TTV might serve as a surrogate for the overall state of the immune system and ability of viral control in immunosuppressed individuals.

#### Mycophenolate Concentration in Blood

Mycophenolic acid was determined with a method modified from Khoschsorur et al. using high performance liquid chromatography (HPLC) (18).

### Statistical Analysis

Patient age was reported as mean and standard deviation. All other continuous variables were summarized as median and interquartile range. Categorical variables were described by frequency and percentage. Differences between groups for continuous and categorical variables were assessed by Wilcoxon rank sum test and Fisher's exact test, respectively. A Wilcoxon rank sum test was used to compare antibody levels between groups. Differences in the number of seroconverted patients and the number of patients not exceeding protective antibody levels were evaluated by means of Fisher's exact test. Changes in individual patients TTV and creatinine levels were assessed by ANOVA with repeated measurements followed by multiple paired Student's *t*-tests. TTV levels were log transformed prior to analysis. *P* < 0.05 were considered significant.

## Results

### Participants

**Figure 1:**
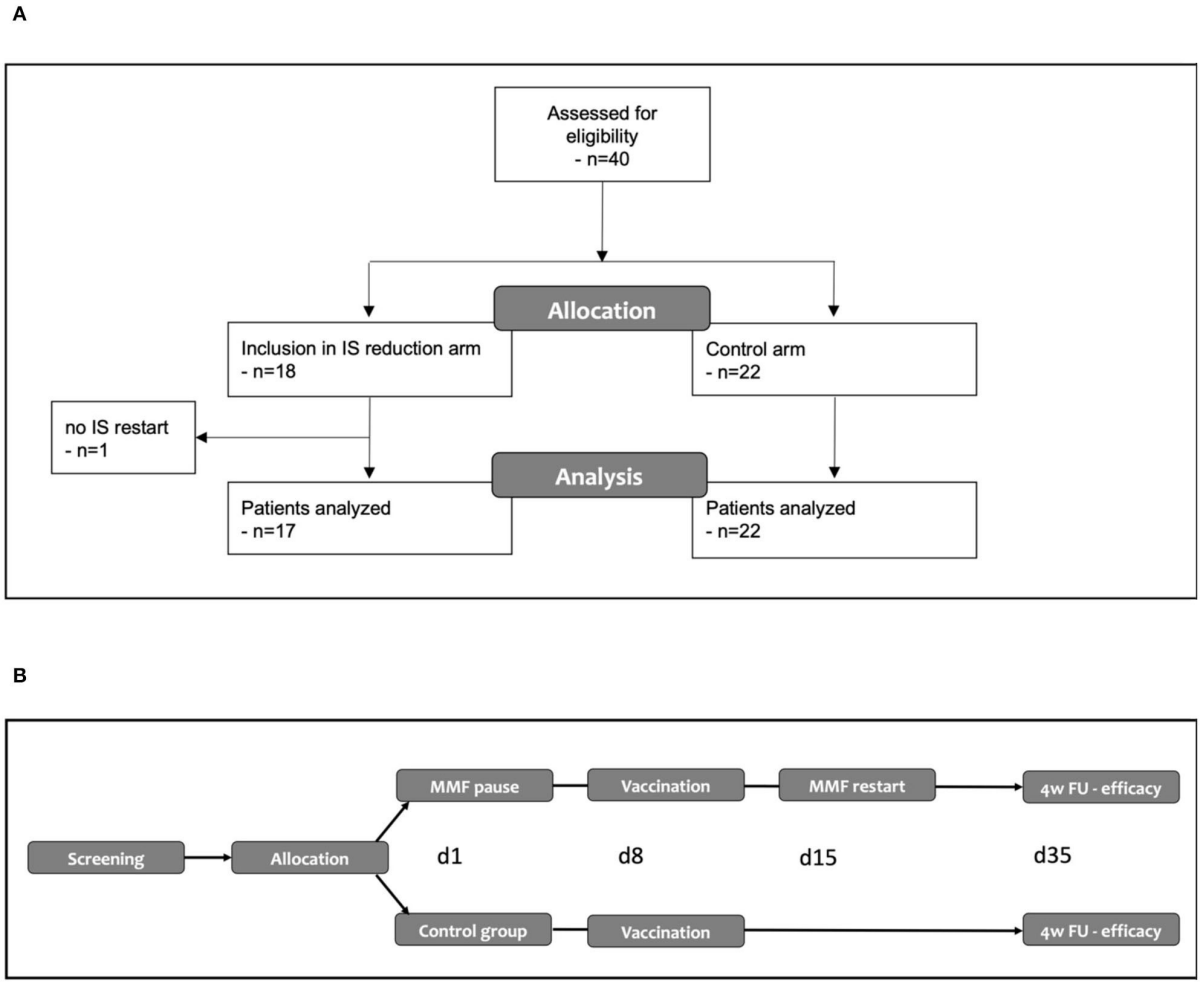
**(A)** Modified CONSORT flow diagram. **(B)** Overview of the study. IS, immunosuppression.

We included a total 40 kidney transplant recipients into this study: MPA/AZA was stopped in 18 patients, while immunosuppressive treatment was not modified in 22 patients serving as control group. The control group included four patients with DSA and three patients with history of biopsy-proven rejection as well as 15 patients who would in principle have been eligible for MPA/AZA discontinuation but opted to receive a 4th vaccination without immunosuppression reduction. In one patient, MPA was not restarted on day 15 due to a concomitant cytomegalovirus (CMV) reactivation. Therefore, only 39 patients were available for the analysis of the primary endpoint (Figures 1A,B).

Patient demographics for these 39 patients are provided in Table 1. With exception of the number of previously administered vaccine doses there were no statistically significant differences between the two groups: In the MPA/AZA pause group 71% of patients had three previous vaccinations and the remaining patients in this group had four previous vaccinations, while all patients in the control group had received three previous vaccine doses. The majority of patients in both groups were on a maintenance immunosuppression regimen with calcineurin inhibitors, MPA and steroids (88 and 86% for immunosuppression reduction and control group, respectively).

**Table 1:** Baseline characteristics.

	**All (*N* = 39)**	**MPA pause (*N* = 17)**	**Control (*N* = 22)**	***P*-value**
Age (SD)	63.97 (9.16)	62.76 (9.18)	64.91 (9.24)	0.372
Sex (female)	15 (38)	4 (24)	11 (50)	0.112
Number of previous vaccine doses				0.011
3	34 (87)	12 (71)	22 (100)	
4	5 (13)	5 (29)	0 (0)	
Time since KTX (years)	3.92 [2.71, 7.54]	4.17 [3.33, 7.75]	3.38 [1.85, 7.27]	0.123
Number of KTX				0.896
1	31 (79)	13 (76)	18 (82)	
2	5 (13)	3 (18)	2 (9)	
3	2 (5)	1 (6)	1 (5)	
5	1 (3)	0 (0)	1 (5)	
Donor type (living)	5 (13)	3 (18)	2 (9)	0.636
Immunosuppression				0.594
Bela, MPA, steroids	4 (10)	1 (6)	3 (14)	
CyA, MPA, steroids	1 (3)	0 (0)	1 (5)	
Tac, MPA, steroids	33 (85)	15 (88)	18 (82)	
Tac, AZA, steroids	1 (3)	1 (6)	0 (0)	
MPA dose (mg/d)	1,000 [812.5, 1,875]	1,000 [720, 1,625]	1,040 [1,000, 1,875]	0.630
DSA>1,000 MFI	4 (11)	0 (0)	4 (20)	0.109
TTV 10^3^ copies/mL	440 [18, 4,600]	195 [9.48, 1,650]	820 [33, 6,000]	0.297
Creatinine (mg/dL)	1.58 [1.37, 2.06]	1.67 [1.54, 1.93]	1.53 [1.21, 2.12]	0.411
Days since last vaccination	136 [127, 141]	139 [65, 140]	135 [129, 142]	0.744
Days vaccination to follow-up	32 [28, 38]	28 [28, 32]	33 [28, 42]	0.317

*Bela, belatacept; MPA, mycophonic acid; CyA, cyclosporin A; Tac, tacrolimus*.

### Rate of Seroconversion

**Figure 2:**
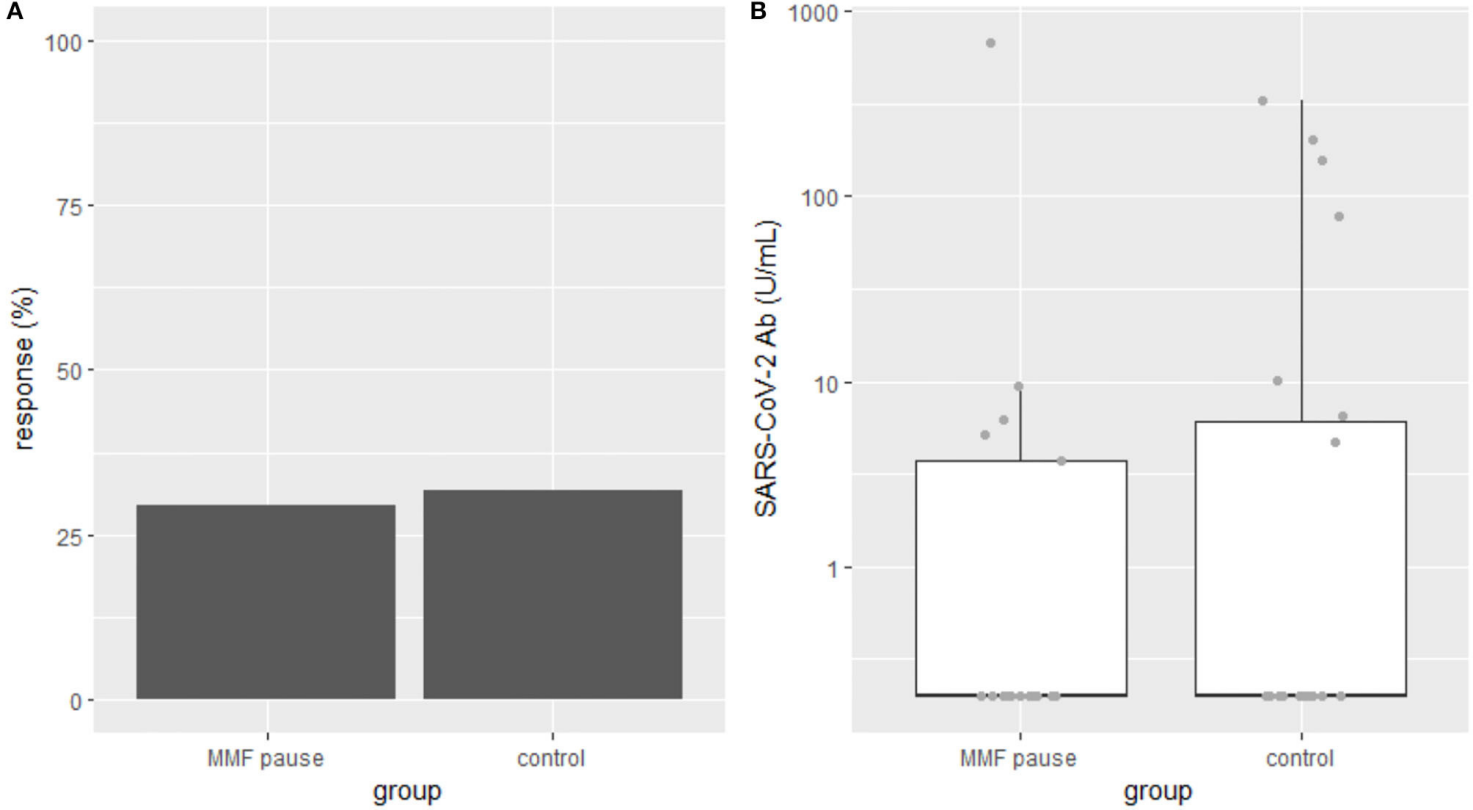
**(A)** Seroconversion rate: Bar chart comparing response rates in KTR with MPA pause and control group following 4th/5th SARS-CoV-2 vaccination with BNT162b2. Response to vaccination was defined by reaching antibody levels above 0.8 U/mL on the Roche Elecsys platform. **(B)** Antibody levels following vaccination: Boxplots comparing antibody levels between MPA pause and control group.

At 4 weeks after vaccination, 29% of patients in the MPA/AZA pause group had detectable SARS-CoV2 spike protein antibodies of >0.8 U/ml (Figure 2A). Due to the imbalance in previous vaccine doses between groups, we then performed separate analyses for individuals with either three or four previous vaccinations: The response rate in the MPA/AZA pause group was 33 and 20% for 4th and 5th vaccination, respectively.

In comparison, the antibody response rate in the control group (all receiving a 4th vaccine dose) was 32% (*p* > 0.99). To address potential imbalances based on the eligibility criteria for MPA/AZA discontinuation, we performed an additional analysis only including patients without DSA or history of rejection who received a fourth vaccination without immunosuppression reduction (response rate 31%).

### Secondary Endpoints

At 4 weeks after vaccination less than one third of patients from each group had antibody levels above limit of detection (0.4 U/mL) and antibody levels were not statistically significantly different between the two groups (*p* = 0.716). From those patients who seroconverted 80% and 86% (*p* > 0.99) had antibody levels below 264 U/mL in the intervention and control group, respectively (Figure 2B).

MPA serum levels measured at the end of the MPA pause were negative for MPA in 16 out of 17 patients, confirming that MPA was held as defined in the protocol. In the one patient with positive MPA serum levels at the end of the MPA pause, MPA level (1.65 μg/mL) was, however, strongly reduced compared to baseline (6.31 μg/mL).

**Figure 3:**
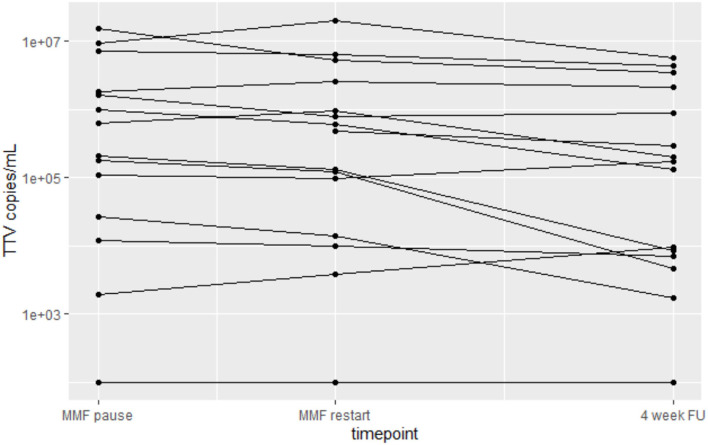
Changes in TTV load in patients at baseline, after 2 weeks of MPA pause and at the 4-week follow-up after vaccination.

Patient individual TTV levels did change following 2-week MPA pause over time (*p* = 0.021). At 4 weeks after vaccination patient individual TTV levels were significantly lower than levels at baseline and at time of MPA restart (Figure 3).

### Safety

No safety signals have been observed in the MPA/AZA pause group. Patient individual serum creatinine levels remained stable throughout the study period (*p* = 0.473). Mean differences in individual patient serum creatine between baseline and 1 week after vaccination and baseline and 4 weeks after vaccination were 0.024 and −0.055 mg/dL, respectively. Donor-derived cell-free DNA as a measure of allograft injury was not detectable in any of the patients at 4 weeks and 8 weeks after MPA/AZA discontinuation. There were no cases of *de novo* donor-specific antibodies 4 week after vaccination.

## Discussion

The main finding of our study was that a 2 week hold of MPA or AZA peri-vaccination did not increase the rate of seroconversion in KTR without response to three or four previous SARS-CoV2 vaccine doses. Interestingly, the observed overall seroconversion rate of 29% in our study cohort was comparable to response rates following initial prime-boost vaccination in KTR or subsequent 3rd vaccine dose in non-responders reported at 36–54 and 30–40%, respectively (2–6). Antibody levels in responders, however, were well below previously published thresholds associated with neutralizing capacity in most patients (14).

These findings are in contrast to the results of two previously published studies investigating the effect of withholding MPA on SARS-Cov2- vaccine response: Discontinuation of MPA for 1 week after, and a variable amount of time before vaccination resulted in a response rate of 92% in a small case series including 24 patients with rheumatic disease compared to 65% in a comparator group including 171 patients who continued therapy (12). However, the concomitant immunosuppression in these patients was not comparable to KTR: In one third of the patients MPA was the only immunosuppressant, and only another third of the patients had a concurrent therapy with prednisone.

Schrezenmeier et al. reported that withholding MPA for 5 weeks around a 4th vaccination in KTR resulted in a much higher seroconversion rate of 76% after 4 weeks, and even 84% in patients on a CNI-based immunosuppressive regimen (19). There are several differences in the study population and intervention: MPA pause in our study was much shorter (2 vs. 5 weeks). Interestingly, when looking at the seroconversion rate 1 week after vaccination and therefore after a comparable duration of MPA pausing, there was already a slightly increased response rate of 34% compared to 24% in our study population. Still, in the majority of patients detectable antibody levels seem to have developed at a later time point (i.e., 1–4 weeks after vaccination) suggesting that prolonged pausing of antiproliferative treatment is required for a sustained response to vaccination. Another factor possibly contributing to the difference in seroconversion between the two studies, however, are the different assays used for measuring antibodies and subsequent different definitions of non-responders. We used a threshold of <0.8 U/ml to identify the non-responders included in our study. Application of a different, less sensitive assay could result in misclassification of patients as non-responders despite low-level antibody responses that can be boosted by additional vaccination. This underlines the importance of a control group, as direct comparison of seroconversion rates across different studies is impaired by different definitions of vaccine non-responders (i.e., sensitivity of antibody detection platform and different cut-off levels for humoral response).

Limitations of our pilot study include the small samples size and the open-label trial design. However, the observed response rates in both groups were virtually identical. Another limitation of the study is the non-randomized trial design, but the control group was comparable in most relevant risk factors for vaccine non-response including levels of immunosuppression and time after transplantation. However, there were some relevant differences between groups: Only 24% of participants in the immunosuppression group were female compared to 50% in the control group. Based on the inclusion criteria for MPA/AZA discontinuation, patients in the control group included individuals with DSA and history of biopsy-proven rejection while the intervention group did not. Availability of alternative COVID-19 prevention strategies (i.e., monoclonal antibodies as prophylaxis) may have influenced patient decision on MPA/AZA pause weighing the benefits of a potentially higher rate of SARS-CoV-2 seroconversion following immunosuppression reduction and the risk for allo-sensitization as consequence of temporary immunosuppression non-adherence. Both groups also differed by the number of previous vaccinations as five individuals in the MPA stop group had received four previous vaccine doses. We, however, observed similar response rates in subgroup analyses only including individuals with three previous vaccine doses in the MPA/AZA pause group or individuals without DSA or history of rejection in the control group. Another limitation of our trial, is the short overall follo-up of only 4 weeks after the additional vaccine dose. As per center policy, all patients without SARS-CoV-2 antibody levels >264 BAU/mL at 4 weeks after the 4th/5th vaccination were subsequently offered pre-exposition prophylaxis with SARS-CoV-2-specific monoclonal antibodies (i.e., sotrovimab or tixagevimab/cilgavimab) impeding further analysis of antibody trajectories beyond 4 weeks.

Definite conclusion on the efficacy of MPA/AZA pause on seroconversion cannot be drawn from this trial due to the non-randomized pilot trial design, the small sample size, and the only short discontinuation of immunosuppression for 2 weeks. The virtually identical response rates across all subgroups, however, suggest that pausing MPA/AZA for 2 weeks discontinuation peri-vaccination only has a limited impact on the seroconversion rate following SASRS-CoV-2 vaccination.

## Conclusion

MPA or AZA discontinuation for 2 weeks peri-vaccination did not result in a higher overall seroconversion rate to an additional vaccine dose compared to a control group without immunosuppression reduction. Overall, one in three KTR without response to previous vaccine doses developed SARS-CoV-2 spike protein antibodies following additional vaccination.

## Data Availability Statement

The raw data supporting the conclusions of this article will be made available by the authors, without undue reservation.

## Ethics Statement

The studies involving human participants were reviewed and approved by Ethics Committee of the Medical University of Vienna. The patients/participants provided their written informed consent to participate in this study.

## Author Contributions

FR, AH, and RR-S conceptualized the trial and wrote the manuscript. KH, LR, FE, IF, SZ, GAB, GB, GF, and RO contributed to data acquisition, data analysis, and writing of the manusript. All authors contributed to the article and approved the submitted version.

## Funding

This study was supported by the Medical-Scientific Fund of the Mayor of the Federal Capital of Vienna (# 21182), the Christine Vranitzky-Stiftung research grant 2020 (both to RR-S), and the Medical University of Vienna Transplantation Research Platform's Start-Up Grant 2020 (to AH).

## Conflict of Interest

The authors declare that the research was conducted in the absence of any commercial or financial relationships that could be construed as a potential conflict of interest.

## Publisher's Note

All claims expressed in this article are solely those of the authors and do not necessarily represent those of their affiliated organizations, or those of the publisher, the editors and the reviewers. Any product that may be evaluated in this article, or claim that may be made by its manufacturer, is not guaranteed or endorsed by the publisher.

## References

[1] Caillard S Anglicheau D Matignon M Durrbach A Greze C Frimat L. An initial report from the French SOT COVID Registry suggests high mortality due to COVID-19 in recipients of kidney transplants. Kidney Int. (2020) 98:1549–58. 10.1016/j.kint.2020.08.005 32853631 PMC7444636

[2] Grupper A Rabinowich L Schwartz D Schwartz IF Ben-Yehoyada M Shashar M. Reduced humoral response to mRNA SARS-CoV-2 BNT162b2 vaccine in kidney transplant recipients without prior exposure to the virus. Am J Transplant. (2021) 21:2719–26. 10.1111/ajt.16615 33866672 PMC8250589

[3] Boyarsky BJ Werbel WA Avery RK Tobian AAR Massie AB Segev DL. Antibody response to 2-Dose SARS-CoV-2 mRNA vaccine series in solid organ transplant recipients. JAMA. (2021) 325:2204. 10.1001/jama.2021.7489 33950155 PMC8100911

[4] Rozen-Zvi B Yahav D Agur T Zingerman B Ben-Zvi H Atamna A. Antibody response to SARS-CoV-2 mRNA vaccine among kidney transplant recipients: a prospective cohort study. Clin Microbiol Infect. (2021) 27:1173. e1. 10.1016/j.cmi.2021.04.028 33957273 PMC8091803

[5] Benotmane I Gautier G Perrin P Olagne J Cognard N Fafi-Kremer S. Antibody response after a third dose of the mRNA-1273 SARS-CoV-2 vaccine in kidney transplant recipients with minimal serologic response to 2 doses. JAMA. (2021) 326:1063–5. 10.1001/jama.2021.12339 34297036 PMC8456389

[6] Reindl-Schwaighofer R Heinzel A Mayrdorfer M Jabbour R Hofbauer TM Merrelaar A. Comparison of SARS-CoV-2 antibody response 4 weeks after homologous vs heterologous third vaccine dose in kidney transplant recipients: a randomized clinical trial. JAMA Intern Med. (2021) 182:165–71. 10.1001/jamainternmed.2021.7372 34928302 PMC8689434

[7] Tau N Yahav D Schneider S Rozen-Zvi B Abu Sneineh M Rahamimov R. Severe consequences of COVID-19 infection among vaccinated kidney transplant recipients. Am J Transplant. (2021) 21:2910–2. 10.1111/ajt.16700 34043872 PMC8222865

[8] Reischig T Kacer M Vlas T Drenko P Kielberger L Machova J. Insufficient response to mRNA SARS-CoV-2 vaccine and high incidence of severe COVID-19 in kidney transplant recipients during pandemic. Am J Transplant. (2021) 22:801–12. 10.1111/ajt.16902 34860470 PMC9906453

[9] Kantauskaite M Müller L Kolb T Fischer S Hillebrandt J Ivens K. Intensity of mycophenolate mofetil treatment is associated with an impaired immune response to SARS-CoV-2 vaccination in kidney transplant recipients. Am J Transplant. (2021) 22:634–9. 10.1111/ajt.16851 34551181 PMC8653081

[10] Allison AC Eugui EM. Purine metabolism and immunosuppressive effects of mycophenolate mofetil (MMF). Clin Transplant. (1996) 10:77–84. 8680053

[11] Maltzman JS Koretzky GA. Azathioprine: old drug, new actions. J Clin Invest. (2003) 111:1122–4. 10.1172/JCI200318384 12697731 PMC152947

[12] Connolly CM Chiang TP-Y Boyarsky BJ Ruddy JA Teles M Alejo JL. Temporary hold of mycophenolate augments humoral response to SARS-CoV-2 vaccination in patients with rheumatic and musculoskeletal diseases: a case series. Ann Rheum Dis. (2022) 81:293–95. 10.1136/annrheumdis-2021-221252 34556484 PMC11034709

[13] Pascual J Van Hooff JP Salmela K Lang P Rigotti P Budde K. Three-year observational follow-up of a multicenter, randomized trial on tacrolimus-based therapy with withdrawal of steroids or mycophenolate mofetil after renal transplant. Transplantation. (2006) 82:55–61. 10.1097/01.tp.0000225806.80890.5e 16861942

[14] Feng S Phillips DJ White T Sayal H Aley PK Bibi S. Correlates of protection against symptomatic and asymptomatic SARS-CoV-2 infection. Nat Med. (2021) 27:2032–40. 10.1101/2021.06.21.21258528 34588689 PMC8604724

[15] Fae I Wenda S Murakösy G Hötzenecker K Jaksch P Fischer GF. Kinetics of donor specific cell free DNA levels in recipients' plasma after lungs transplantation. HLA. (2020) 95:279. 10.1111/tan.13844

[16] Eskandary F Regele H Baumann L Bond G Kozakowski N Wahrmann M. A randomized trial of bortezomib in late antibody-mediated kidney transplant rejection. J Am Soc Nephrol JASN. (2018) 29:591–605. 10.1681/ASN.2017070818 29242250 PMC5791086

[17] Doberer K Schiemann M Strassl R Haupenthal F Dermuth F Görzer I. Torque teno virus for risk stratification of graft rejection and infection in kidney transplant recipients-A prospective observational trial. Am J Transplant. (2020) 20:2081–90. 10.1111/ajt.15810 32034850 PMC7496119

[18] Khoschsorur G Erwa W. Liquid chromatographic method for simultaneous determination of mycophenolic acid and its phenol- and acylglucuronide metabolites in plasma. J Chromatogr B Analyt Technol Biomed Life Sci. (2004) 799:355–60. 10.1016/j.jchromb.2003.10.074 14670756

[19] Schrezenmeier E Rincon-Arevalo H Jens A Stefanski A-L Hammett C Osmanodja B. Temporary antimetabolite treatment hold boosts SARS-CoV-2 vaccination-specific humoral and cellular immunity in kidney transplant recipients. JCI insight. (2022) 7:e157836. 10.1172/jci.insight.157836 35349490 PMC9090237

